# Fortuitous discovery of an anomalous connection of the coronary arteries during an urgent coronary angiography: a case report

**DOI:** 10.11604/pamj.2021.39.45.29216

**Published:** 2021-05-18

**Authors:** Amine Ech-chenbouli, Mohamed Elghali Benouna, Salim Arous, Rachida Habbal

**Affiliations:** 1 Cardiology Department Ibn Rochd University Hospital, Internat Elfaidouzi, University Hospital Center Ibn Rochd, Casablanca, Morocco

**Keywords:** Cardiac computed tomography, coronary angiogram, a case report

## Abstract

Anomalous connection of the coronary arteries (ANOCOR) is a rare angiographic finding; although most ANOCORs are benign some are a risky condition that can lead to sudden death. Knowing their particularities is important to know how to manage them. With an angiographic prevalence averaging 1%, proximal anomalous connection of the Coronary arteries (ANOCOR) represents a diverse entity. The challenge is to distinguish the benign ANOCOR to reassure the patient from the high risk ANOCOR that may need surgical repair. We present a case of a 52-years-old man admitted to our cath lab for urgent coronary angiography after a non ST segment elevation myocardial infarction. At coronary angiography we found ectopic left anterior descending and circumflex arteries with a culprit lesion on the right non ectopic coronary artery. A computed tomography (CT) scan showed benign ectopic courses of the left anterior descending and circumflex arteries. No surgical repair was indicated.

## Introduction

With an angiographic prevalence averaging 1% [1], proximal anomalous connections of the coronary arteries (ANOCOR) represent a diverse entity. Beside frequent anatomical variations, the main ANOCORs are the connection in a contra-lateral sinus or a contra-lateral artery, the connection in a normal sinus but in an eccentric position, the aortic connection abnormally high, the single coronary artery and the connection of the left coronary artery with the pulmonary artery. Whereas most of ANOCORs are considered as benign abnormalities, a small number of ANOCORs can be associated with a risk of sudden cardiac death. The challenge is to distinguish the benign ANOCOR to reassure the patient from the high risk ANOCOR that may need surgical repair. Through this article we will present a case of a fortuitous discovery of an ANOCOR of a left anterior descending and circumflex arteries which represent only 0.05% [1] of total angiography findings.

## Patient and observation

**Figure 1:**
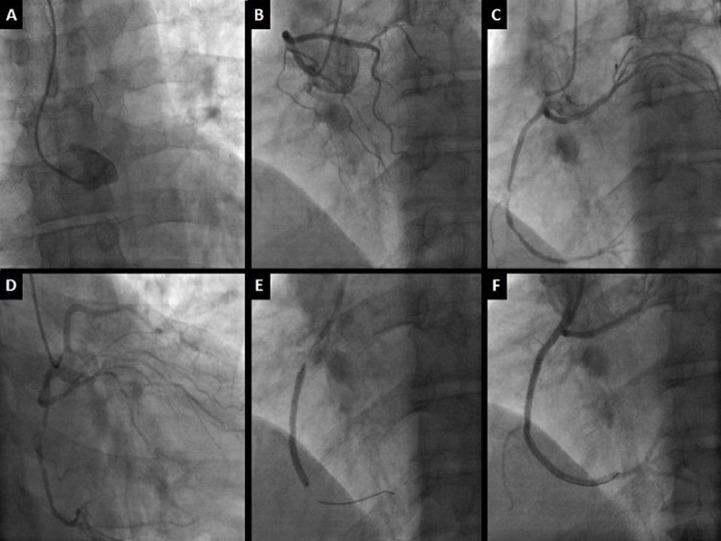
coronary angiography images showing: left main artery not visualised in the left sinus (panel A); left anterior descending artery connected to right sinus (panel B); circumflex artery connected to right coronary artery (panel C); very tight stenosis of the right middle coronary artery (panel D); drug eluting stent positioned at the level of the right middle coronary artery (panel E); final result after angioplasty (panel F)

**Figure 2:**
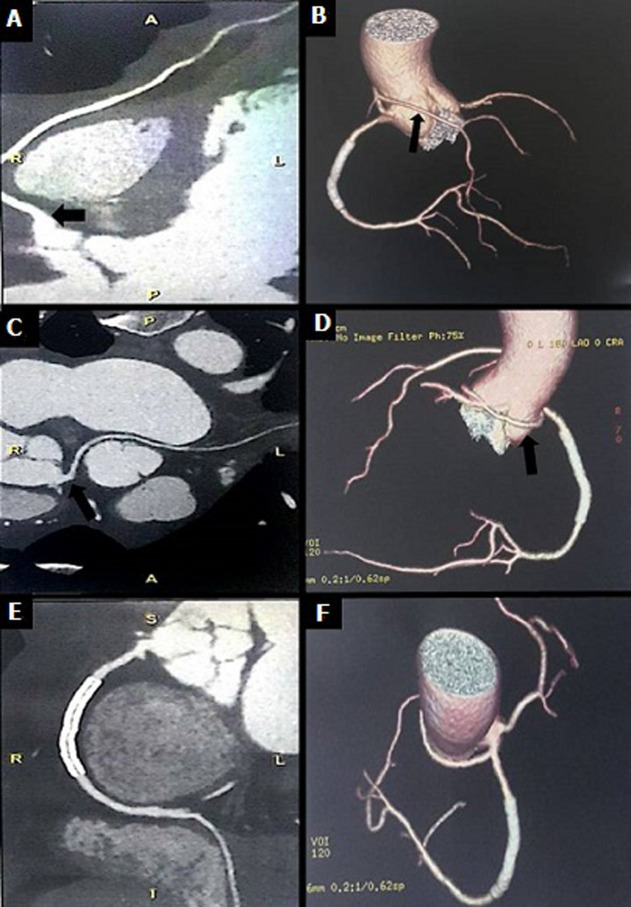
coronary computed tomography scan with 3D reconstruction showing: ectopic left anterior descending artery (black arrow) connected to the right sinus with a prepulmonary course (panel A and B); ectopic circumflex artery (black arrow) connected to the right coronary artery with a retroaortic course (Panel C and D), non ectopic right coronary artery with a permeable stent (Panel E and F)

A 52-years-old man was admitted to the emergency department for chest pain evolving since two days. The patient had no history of coronary disease, diabetes or smoking habits. On clinical examination, the patient, had a heart rate of 88 b.p.m. a blood pressure of 110/80 mmHg, normal heart sounds on auscultation; he had no dyspnea or signs of heart failure. His electrocardiogram (EKG) showed an ST segment depression on inferior leads; blood tests objectified Troponin-IC elevation. An urgent transradial coronary angiography was then realized objectifying empty left sinus and an ectopic left anterior descending artery connected to the rights sinus and circumflex artery connected to the right coronary artery; the injection of the right coronary artery showed a severe stenosis on its middle segment (Figure 1). The patient had successful angioplasty with a 3.0 x 38 milimetre drug eluting stent and was put under a one year double antiaplatelet therapy regimen with Clopidogrel 75 mg per day and Aspirin 75 mg per day. After the angioplasty we had to manage the anomalous connection of the coronary arteries to seek for high risk futures. The patient had a coronary computed tomography that showed an ectopic left anterior descending artery connected to the right sinus above the right coronary artery with a pre-pulmonary course, an ectopic circumflex artery connected to the proximal right coronary artery with a retro aortic course and a non-ectopic right coronary artery with a permeable stent (Figure 2).

## Discussion

### What are the most frequent ANOCORs?

ANOCORs with a connection in the contra lateral sinus or artery are the most frequent (nearly 90%), while the connections in the non-coronary sinus or in the pulmonary artery remain exceptional, as well as single coronary arteries [1]. Abnormally high connections, located in the ascending aorta more than 10 mm from the Sino-tubular junction remain rare. The circumflex artery is involved in almost half of cases (47.4%) and the right coronary artery in a third of cases (33.3%). Left main (12.1%) and left anterior descending artery (5.4%) are less often affected [1]. The other important anatomical point to consider is the initial ectopic course of the ANOCOR, defined between the abnormal connection and the point where the artery joins a usual myocardial area. It's important to know that some courses are more frequent depending on the coronary artery involved: The initial course of an ectopic circumflex is almost exclusively retro-aortic, a right ANOCOR is associated with a pre-aortic path in nearly 90% cases, the left main and left anterior descending artery can be associated with all types of courses, specifying that the retro-pulmonary course is the most frequent (almost one case in two) and that the pre-aortic pathway is very rare (close to 5% of case) [1].

### Is there a relationship between coronary syndrome and ANOCORs?

The question in front of such an association is whether the congenital anomaly may have played a role in the occurrence of the coronary syndrome. It is then necessary to distinguish the site of the lesion responsible for the coronary syndrome, either it happened on the ectopic coronary segment as defined above, or it sits on the non-ectopic segment [2]. In our case, we are in front of a usual non ST segment elevation myocardial infarction with a fortuitous discovery of an ANOCOR. This situation is quite frequent and it could raise the question of a possible secondary correction of an ANOCOR with high anatomical risk. The responsibility for ANOCORs with pre-aortic path is established in the occurrence of sudden death [3]. The usual profile is a young subject (< 35 years) without any cardiovascular history. Serious cardiac events (sudden death, syncope) generally occur during (or just after) intense physical exertion and more particularly in sport practice [4]. Analysis by endocoronary ultrasound of ANOCOR with an intramural pathway and fortuitous discovery at an age > 35 years suggests that an intramural pathway would have little or no risk of atherosclerosis [5]. The mechanism of a possible protective role of constituents of the arterial wall remains unknown. A situation inverse is suggested for the ectopic segment of an ANOCOR involving the circumflex artery. Some studies have shown an increased prevalence of coronary atheroma in an ectopic circumflex artery [6]. It should be noted that the samples studied were often small and that the angiographic analysis did not necessarily distinguished between ectopic segments and non-ectopic segments. Logically, the prevalence of atherosclerotic disease on non-ectopic segments should identical to that of a population exposed to classic risk factors [2].

### What are the abnormalities at risk?

Apart from an associated atheromatous disease, often revealing the anomaly after the age of 40, the severity of certain anatomical forms is well established while the large majority of ANOCORs are generally considered to be benign. Among these, we can cite the forms with an initial pre-pulmonary or retro-aortic course. In the other hand, an initial pre-aortic course must always be taken into consideration. The pre-aortic course mainly concerns the left main or the left anterior descending artery, but also the right coronary. The frequency of a pre aortic course is higher for the right coronary than for the left coronary, due to a connection often close to the anterior commissure for the right coronary artery [6].In an autopsy series of 242 congenital coronary anomalies, 49 ectopic left main connections were individualized from the right sinus and 52 ectopic connections of the right coronary artery from the left sinus [7]. In this series, 57% of left ANOCORs and 25% of right ANOCORs were associated with sudden death, with in the vast majority of cases the presence of a pre-aortic course. The particularity of this pathway is its mode of discovery, often sudden death occurring in an adolescent or young adult during exertion usually violent. The prognosis for an ANOCOR discovered before or after the age of 40 is different, even if it is a risky form. In a series of 690 sudden deaths occurring between the ages of 14 and 40, the prevalence of a coronary anomaly as the identified cause of death is 8% between 14 and 20 years, 4% between 21 and 30 years and only 0.5% between 31 and 40 years [7].

### What to do with an ANOCOR at risk?

The discovery of an ANOCOR must be combined with the determination of its potential severity. For anatomical shapes considered benign, abstention from treatment is the rule. For the anatomical shapes considered to be at risk, we must recognize the current absence of a consensual attitude. The surgical indication relates mainly to abnormal connections of the left main or the left anterior descending artery with a pre-aortic initial course [8]. The method of choice is to perform a lateral anastomosis of the ANOCOR in the left sinus, thus creating a new ostium. Surgical repair is a lot more rarely offered in case of right ANACOR [9].

## Conclusion

Discovery of an ANOCOR while performing a coronary angiography is a relatively rare event. The Knowledge of the main ANOCORs and their particularities is very important to manage these situations. CT scan remain a valuable tool to precise the initial course of the ANOCOR, the relationship between the ANOCOR and the principal vessels and the existence of an aortic intramural pathway to establish the risk of sudden death.
